# Abnormal vibration perception threshold alters the gait features in type 2 diabetes mellitus patients

**DOI:** 10.3389/fendo.2022.1092764

**Published:** 2023-02-08

**Authors:** Lining Dong, Yanyun Hu, Lei Xu, Hui Zeng, Wenqi Shen, Patrick Esser, Helen Dawes, Fang Liu

**Affiliations:** ^1^ Department of Endocrinology and Metabolism, Shanghai General Hospital, Shanghai Jiao Tong University School of Medicine, Shanghai, China; ^2^ Shanghai Jiao-Tong University Affiliated Sixth People’s Hospital, Shanghai Clinical Medical Center of Diabetes, Shanghai Key Clinical Center of Metabolic Diseases, Multi-disciplinary Collaboration Diabetic Foot Group, Shanghai, China; ^3^ Faculty of Health and Life Sciences, Oxford Brooks University Affiliated Movement Science Institute, Headington, United Kingdom; ^4^ Department of Public Health and Sports Sciences, Faculty of Health and Life Sciences, College of Medicine, University of Exeter, Exeter, United Kingdom

**Keywords:** gait, diabetic peripheral neuropathy, vibrating perception threshold, diabetic, complication, type 2 diabetes

## Abstract

**Objective:**

It is generally believed that gait characteristics of diabetic neuropathic patients differ from those of non-diabetic ones. However, it is still unclear how the abnormal foot sensation influences the gait during walking in type 2 diabetes mellitus (T2DM). For the purpose of gaining a better insight into the alterations of detailed gait parameters and figuring out important aspects in the gait indexes by peripheral neuropathy in elder T2DM patients, we compared the gait features in participants with normal glucose tolerance (NGT) controls and diabetic individuals complicated by peripheral neuropathy or not.

**Subjects and methods:**

Gait parameters were observed during the 10-m walk on flat land among different conditions of diabetes in 1,741 participants from three clinical centers. Subjects were divided into four groups: persons with NGT were taken as the control group; patients with T2DM included three subgroups: DM control (no chronic complications), DM-DPN (DM complicated by only peripheral neuropathy), and DM-DPN+LEAD (DM complicated by both neuropathy and artery disease). The clinical characteristics and gait parameters were assessed and compared among these four groups. Analyses of variance were employed to verify possible differences of gait parameters between groups and conditions. Stepwise multivariate regression analysis was performed to reveal possible predictors of gait deficits. Receiver operating characteristic (ROC) curve analysis was employed to find any discriminatory power of diabetic peripheral neuropathy (DPN) for the step time.

**Results:**

In participants burdened with DPN, whether complicated by lower extremity arterial disease (LEAD) or not, step time increased sharply (*p* < 0.05). Stepwise multivariate regression models showed that independent variables of gait abnormality were sex, age, leg length, vibration perception threshold (VPT), and ankle-brachial index (ABI) (*p* < 0.01). Meanwhile, VPT was a significant independent predictor of step time, spatiotemporal variability (SD_A_), and temporal variability (SD_B_) (*p* < 0.05). ROC curve analysis was explored to find the discriminatory power of DPN for the occurrence of increased step time. The area under the curve (AUC) value was 0.608 (95% CI: 0.562–0.654, *p* < 0.01), and the cutoff point was 538.41 ms accompanied by a higher VPT. A significant positive association was observed between increased step time and the highest VPT group [odds ratio (OR) = 1.83, 95% CI: 1.32–2.55, *p<* 0.01]. In female patients, this OR value elevated to 2.16 (95% CI: 1.25–3.73, *p<* 0.01).

**Conclusions:**

In addition to sex, age, and leg length, VPT was a distinct factor that associated with altered gait parameters. DPN is associated with increased step time, and the step time increases with worsening VPT in type 2 diabetes.

## Introduction

Gait analysis provides an objective means of measuring walking (1) and presents biomechanical differences depending on individual characteristics, such as morphological nature, physical activity, age, and the presence of some diseases. In a recent report, an altered gait pattern is apparently observed among individuals with diabetic peripheral neuropathy (DPN) or other diabetic complications, even in diabetes alone, including slower gait speed, shorter stride length, increased cadence, and high gait variability (2–6).

It is generally believed that up to 50% of people with diabetes will develop significant peripheral neuropathies (7). The presence of DPN, leading to an increased number of repetitive falls compared with individuals without diabetes (7), also significantly reduces walking ability and causes the alterations of foot posture and function (8) and could cause abnormal gait during walking. Several recent studies implicated that the main abnormalities in gait parameters among DPN include decreased walking speed, shorter steps, and greater variability of step timing (9), exhibiting a more conservative gait pattern, which resulted from peripheral sensory loss rather than from vision deficiency or decreased lower-limb muscle strength, and the differences were particularly evident on an irregular surface. However, different studies yielded controversial results because of various examining devices and diverse subjects. For instance, de Mettelinge et al. (10) reported that gait patterns did not differ significantly between diabetes complicated by neuropathy and diabetes not complicated by neuropathy.

Although most emerging evidence focused on the association of specific DPN and foot plantar pressure, understanding the effects of other diabetic conditions on gait still showed their importance, and a wealth of studies have been designed to investigate their possible relationships. It has been well documented that the gait pattern can be dramatically altered in persons with diabetes, including slowed gait speed, shorter steps, prolonged double support time, and increased step width, as well as gait variability (11). However, a cross-sectional study of diabetes mellitus (DM) patients with DPN (n = 20), without DPN (n = 26), and age-/gender-/Body Mass Index (BMI)-matched healthy control subjects (n = 20) that was conducted by Yavuzer et al. (12) showed that diabetic patients with DPN had slower gait, shorter steps, limited knee and ankle mobility, and lower plantar flexion moment and power than the healthy control group. There was also a trial that proved no difference between diabetic patients with neuropathy and diabetic patients without neuropathy (10).

Although dictated by the specific matching procedure, the relatively small sample size could be considered as a limitation of these studies. Moreover, a comprehensive study focused on gait characteristics has never been accomplished in healthy controls and patients with type 2 diabetes, DPN, or DPN complicated by lower extremity arterial disease (LEAD) in the same clinical trial. Based on these considerations and those controversies, we believe that it is particularly urgent to explore the impact of different glucose conditions and chronic diabetes complications on gait. Thus, the aim of the study was to determine how gait components were affected by diabetic neuropathy.

## Subjects and methods

### Subjects

A cross-sectional observational study was conducted. All of the individuals were diagnosed as having normal glucose tolerance (NGT) and type 2 diabetes mellitus (T2DM) based on American Diabetes Association 2020 standards (13). DPN was screened and confirmed if the vibration perception threshold (VPT) was >25 volts (V) in combination with a positive Neuropathy Deficit Score (NDS) (14). LEAD was diagnosed if the ankle-brachial index (ABI) was <0.9 (15). For comorbid conditions, inclusion criteria for chronic heart disease (CHD): 1) history of myocardial infarction; 2) coronary stents or coronary artery bypass grafting is excluded. The inclusion criteria for cerebral infarction were as follows: 1) a history of old cerebral infarction; 2) physical activity was not affected by cerebral infarction. Patients with acute cerebral infarction within 3 months and any other cerebrovascular accident were excluded. Other exclusion criteria were the presence of any orthopedic, visual, neurological, or other disturbance that might affect gait, including current pain, injury, a history of diabetic foot, Parkinson’s disease, moderate and severe lumbar disease, active ulceration or amputation and diabetic ketoacidosis, hyperosmolar hyperglycemia syndrome, and other acute diabetic complications. The study was approved by the Ethics Committee of the Shanghai Sixth People’s Hospital (ethical approval number: ChiCRT-DDD-16009531), and written informed consents were obtained from all of the participants.

A total of 1,741 individuals (868 men and 873 women) were enrolled from the Shanghai Clinical Medical Center of Diabetes, the First Affiliated Hospital of Anhui Medical University, and West China Hospital of Sichuan University; 49.9% were men, with a mean age of 60.95 ± 9.25 years. Participants were recruited and assigned into one of four groups: subjects with normal diabetic tolerance (NGT, n = 282); T2DM without peripheral neuropathy or LEAD (DM, n = 1,266); T2DM complicated by only peripheral neuropathy (DPN, n = 144); T2DM with both DPN and LEAD (n = 49).

### Procedures

All subjects’ sex, age, BMI, diabetes duration, hypertension (HP), CHD, and cerebral infarction were collected. BMI was calculated as body weight (in kg) divided by the square of the height (in m). The history of smoking was recorded based on self-report of all subjects. Levels of fasting plasma glucose (FPG) and 2-h postprandial blood glucose (PPG) were estimated by the glucose oxidase method. Glycosylated hemoglobin (HbA1c) was determined by high-pressure liquid chromatography using the VariantÔ II machine (Bio-Rad Inc., Hercules, CA, USA).

A neuropathic assessment of VPT was performed by the same technician using a neurothesiometer (Bio-Thesiometer; Bio-Medical Instrument Co., Newbury, OH, USA). The operational approaches were based upon the International Working Group on the Diabetic Foot of the International Diabetes Federation. The higher value of VPT in the limb was selected for our analysis. The ABI, the ratio of ankle systolic pressure to arm systolic pressure, was performed according to the standard protocols of the International Diabetes Federation. The lower value of ABI in the limb was opted for our analysis.

For the gait data collection, a smart portable wireless gait measurement instrument named gait crasher was provided by our research partners from Brooks University. In brief, a commercially available LPMS (LP-RESEARCH Motion Sensor, 400 Hz, Japan) was attached over the skin of the fourth lumbar vertebra. Participants were asked to walk over a 10-m walkway free of obstacles at their comfortable walking pace. Participants started at a static position at the 0 point, came to a complete stop at the 10-m line. Two successful walks were conducted for each participant. All parameters of the gait cycle are registered and can be analyzed using Vicon 512 Motion Analysis System (Oxford Metrics Ltd., Oxford, England) in great detail. Descriptive statistics of the spatiotemporal gait parameters for both 10-m level walks were calculated and analyzed: cadence, stride length, walking speed, duty-factor_double stance, step time, and walk ratio (step length–cadence ratio). Phase plot description of gait included spatiotemporal variability (SD_A_), temporal variability (SD_B_), A ratio, and symmetry (Δangleβ). SD_A_ means the spatial and temporal variability of the vertical trunk movement during walking but also is influenced by the magnitude of vertical trunk movement. SD_B_ reflects the symmetry of trunk movement from stride to stride. A ratio was described by the ratio between SD_A_ and SD_B_. Δangleβ was calculated as the angle difference between the SD_A_ vector and 45°. We collected and analyzed the spatiotemporal and phase plot variables per group in the middle section of the walkway, avoiding acceleration and deceleration periods during gait.

### Data analysis

Categorical variables were expressed as percentages, and continuous variables were given as mean ± SD values. Comparison of continuous variables among the four groups was performed using one-way analysis of variance (ANOVA). Nonparametric testing was accomplished by the Kruskal–Wallis test. Associations between gait parameters and other variables were evaluated with stepwise multiple regression analysis. Logistic regression analysis was performed to evaluate the odds ratio (OR) and associated factors. The OR (95% CI) was calculated in two logistic regression models: a non-adjusted model and an age-adjusted model. A receiver operating characteristic (ROC) curve was employed to find a cutoff of step time for the presence of DPN. Statistical analyses were performed using SPSS version 24.0 software (SPSS Inc., Chicago, IL, USA). *p* < 0.05 was considered statistically significant.

## Results

### Clinical characteristics

Basic characteristics of the participants were listed in **Table 1**. There were overall significant differences among the four groups in age, sex, height, leg length, and diabetes duration (*p* < 0.01). Although not the tallest, participants with DPN showed the longest leg length (*p* < 0.05). Compared with NGT groups, diabetic participants exhibited a poorer condition of health and living habits; for instance, they were more likely to have HP, CHD, and cerebral infarction (all *p* < 0.05), as well as a higher proportion of smokers (*p* < 0.05). The highest value of VPT (36.43 V) was detected among patients complicated by both DPN and LEAD (*p* < 0.05), followed by DPN individuals. In general, FPG, PPG, and HbA1c gradually increased with the aggravation of the disease (*p* < 0.05), and these groups showed an obvious trend of increasing age, higher prevalence of complications, and relatively worse condition.

**Table 1:** Comparison of basic characteristics among groups with and without diabetes and complications.

	NGT	DM	DM-DPN	DM-DPN+LEAD	*p*
Patients (n,%)	282, 16.2	1,266, 72.7	144, 8.3	49, 2.8	–
Male (n, %)	104, 36.9	639, 50.5	93, 64.6	32, 65.3	<0.001
Age (years)	61.07±7.93	60.41±9.59	64.32±6.37^*^a^^	63.91±9.34^b^	<0.001
Height (cm)	162.52±7.44	164.67±8.19^*^	165.24±8.11^*^a^^	165.52±7.58^*^a^^	<0.001
Leg length (cm)	91.17±4.76	91.93±5.34^*^	93.61±5.69^*^a^^	92.80±5.21^*^a^^	<0.001
BMI (kg/m^2^)	24.16±3.03	24.67±3.31	24.99±3.13	24.19±3.61	0.099
DM duration (years)	–	9.35±7.03	11.18±7.59^a^	14.81±7.11^ab^	<0.001
Smokers (n,%)	20, 7.0	353, 27.9^*^	53, 36.6^*^a^^	23, 46.5^*^ab^^	<0.001
Comorbidities (n,%)					
HP	27.0	46.4^*^	60.7^*^a^^	58.1^*^a^^	<0.001
CHD	1.8	13.2^*^	22.3^*^a^^	14.0^*^b^^	<0.001
Cerebral infarction	3.9	11.1^*^	18.8^*^a^^	4.7^ab^	<0.001
VPT(V)	12.00±1.01	14.30±4.30^*^	31.98±8.83^*^a^^	36.43±13.26^*^ab^^	<0.001
ABI	1.10±0.12	1.12±0.09	1.13±0.11	0.74±0.25^*^ab^^	<0.001
HbA1c (%)	–	7.49±1.60	7.24±0.96	7.52±2.26^ab^	0.252
FPG (mmol/l)	5.50±0.73	8.23±2.41^*^	8.58±2.61^*^a^^	8.90±2.57^*^	<0.001
PPG (mmol/l)	–	11.45±3.55	12.08±4.15	12.58±6.32^ab^	0.076

Data were presented as mean ± SD or n (%) as appropriate.

*p < 0.05 compared with NGT; ^a^p < 0.05 compared with DM; ^b^p < 0.05 compared with DPN. Data marked with the same letter mean no significant difference between groups.

NGT, normal glucose tolerance; VPT, vibration perception threshold; ABI, ankle-brachial index; CHD, chronic heart disease; HP, hypertension; BMI, body mass index; HbA1c, glycosylated hemoglobin; FPG, fasting plasma glucose; PPG, 2-h postprandial blood glucose. DM, diabetes mellitus; DPN, diabetic peripheral neuropathy; LEAD, lower extremity artery disease.

### Alterations of gait parameters among different groups

**Figure 1:**
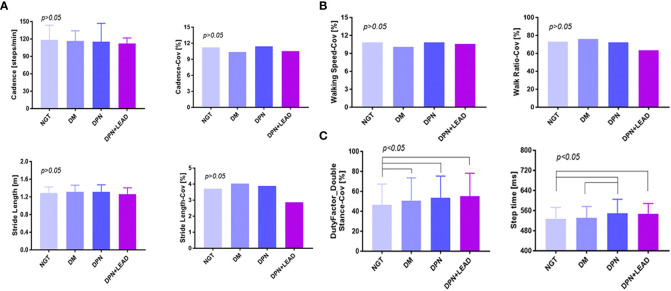
The comparison of gait spatiotemporal variables among different groups. **(A–C)** Duty-factor_double stance-CoV was different between the non-diabetic group and the diabetic subgroups, and the step time was different between the non-diabetic group and the complication groups. Variables were cadence, stride length, walking speed, walk ratio, duty-factor_double stance, step time, and CoVs (CoV = SD/mean x 100). The difference of all gait variables between groups was significant (*p* < 0.05). Subjects with abnormal blood glucose control showed a considerably higher duty-factor_double stance-CoV (52.50% vs. 46.30%, *p* < 0.05). In participants burdened with DPN, whether complicated by LEAD or not, step time increased sharply (*p* < 0.05).

Spatiotemporal analysis was conducted for cadence, stride length, walking speed, walk ratio, duty-factor_double stance, and step time (**Figure 1**). Coefficient of variation (CoV) (SD/mean x 100) was used to assess the variability in these spatiotemporal parameters. As illustrated, no notable differences of cadence, stride length, walking speed, walk ratio, and related CoVs were seen, and no obvious trend was exhibited (all *p* > 0.05) among subjects with all groups. Compared with individuals with abnormal glucose metabolism, subjects with NGT showed a considerably lower duty-factor_double stance-CoV (46.3% ± 1.4% vs. an average of 52.5% ± 0.6%, *p* < 0.05, Figure 1C). In participants burdened with DPN, step time increased sharply (548.7 ± 4.8 ms in DPN vs. 527.7 ± 2.7 ms in NGT vs. 530.6 ± 1.3 ms in diabetes, *p* < 0.05, **Figure 1C**). Furthermore, DPN individuals were subdivided into symptomatic DPN (n = 77, 53.5%) and non-symptomatic DPN (n = 67, 46.5%); no difference was detected between the two groups (544.6 vs. 551.8 ms, *p* > 0.05).

**Figure 2:**
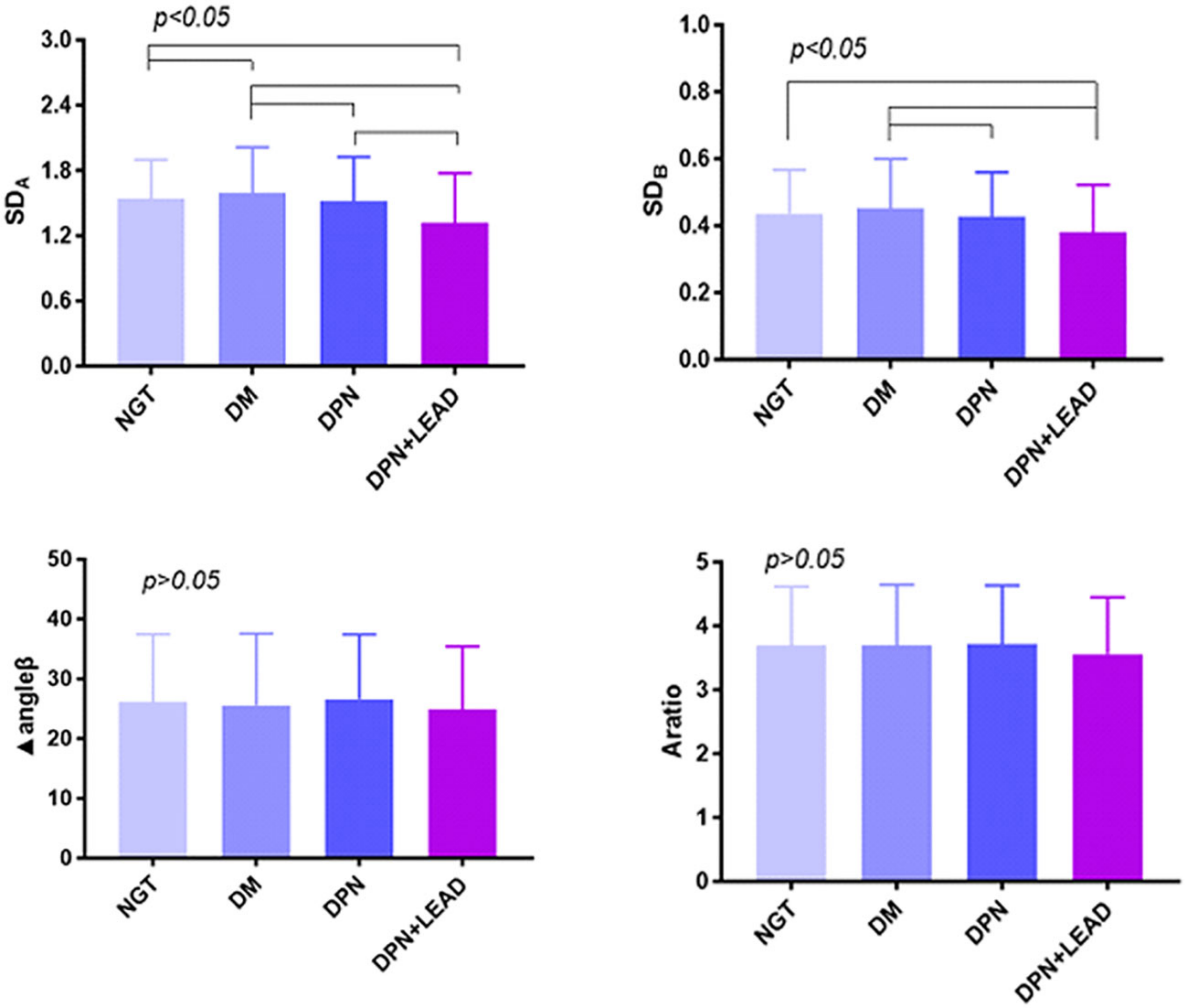
The comparison of gait phase plot variables among the different groups. Variables were SD_A_, SD_B_, A ratio, and Δangleβ. The difference of all gait variables between groups was significant (*p* < 0.05). Trend analysis. The results displayed lower SD_A_ (1.32 vs. 1.57, *p* < 0.01) and SD_B_ (0.38 vs. 0.51, *p* < 0.01) in subjects with both DPN and LEAD. Moreover, trend analysis revealed gradually decreased SD_A_ and SD_B_ in the four DM groups (*p* < 0.05).

Phase plot analysis was performed for SD_A_, SD_B_, A ratio, and Δangleβ (**Figure 2**). The results displayed lower SD_A_ (1.32 ± 0.09 vs. an average of 1.57 ± 0.01 in NGT and DM, *p* < 0.01) and lower SD_B_ (0.38 ± 0.03 vs. an average of 0.51 ± 0.01 in NGT and DM, *p* < 0.01) in subjects with both DPN and LEAD. Moreover, trend analysis revealed that SD_A_ and SD_B_ gradually decreased in the three DM groups (*p* < 0.05). No significant difference was found for A ratio and Δangleβ among the four groups (*p* > 0.05).

### Factors associated with altered gait parameters

Stepwise multivariate regression models used to predict gait characteristics are shown in **Table 2**. For gait parameters, the significant independent variables were sex and/or age (*p* < 0.01). Leg length turned out to be another important index that could influence stride length, walking speed, walk ratio, SD_A_, and SD_B_ (*p* < 0.01). VPT was a significant independent predictor of step time (B = 0.654, *p* = 0.000), SD_A_ (B = -0.007, *p* = 0.005), and SD_B_ (B = -0.002, *p* = 0.000).

**Table 2:** Regression coefficient summary for independent variables included in multivariate regression models for gait characteristic dependent variables–Spatiootemporal analysis and phase plot analysis.

Dependent variables	Predictors	Regression coefficient (B)	95% CI	Adjusted R^2^	*p* value
Spatiotemporal analysis					
Cadence	Sex	6.584	4.415 ~ 8.753	0.049	0.000
(strides/min)	Age	-0.354	-0.448 ~ -0.286	0.034	0.000
	Constant	128.802	122.685 ~ 134.320		
Step time	Sex	-19.681	-25.057 ~ -14.305	0.038	0.000
(ms)	VPT	0.654	0.290 ~ 1.018	0.054	0.000
	Age	0.418	0.132 ~ 0.703	0.058	0.000
	Constant	524.993	507.707 ~ 542.280		
Stride length	Leg length	0.009	0.008 ~ 0.011	0.199	0.000
(m)	Sex	-0.074	-0.092 ~ -0.057	0.242	0.000
	ABI	0.088	0.002 ~ 0.142	0.247	0.002
	Constant	0.460	0.282 ~ 0.639		
Walking speed	Age	-0.004	-0.005 ~ -0.003	0.041	0.000
(m/s)	Leg length	0.009	0.007 ~ 0.011	0.066	0.000
	Constant	0.706	0.478 ~ 0.926		
Walk ratio	Sex	-0.655	-0.739 ~ -0.571	0.175	0.000
(mm/steps/min)	Leg length	0.038	0.030 ~ 0.046	0.206	0.000
	Constant	3.114	2.297 ~ 3.932		
Duty factor	Sex	-1.945	-2.824 ~ -1.066	0.015	0.000
-Double Stance	ABI	-4.135	-7.296 ~ -1.066	0.020	0.010
	Constant	36.794	33.039 ~ 40.548		
Phase plot analysis					
SD_A_	Sex	-0.211	-0.262 ~ -0.160	0.097	0.000
	VPT	-0.007	-0.010 ~ -0.004	0.123	0.005
	Leg length	0.009	0.004 ~ 0.013	0.133	0.007
	Age	-0.004	-0.006 ~ 0.002	0.140	0.011
	ABI	0.204	0.046 ~ 0.363	0.144	0.015
	Constant	1.233	0.696 ~1.769		
SD_B_	Sex	-0.052	-0.070 ~ -0.033	0.044	0.000
	VPT	-0.002	-0.003 ~ -0.001	0.057	0.000
	Leg length	0.002	0.001~ 0.004	0.062	0.000
	ABI	0.059	0.002 ~ 0.116	0.065	0.002
	Constant	0.275	0.090 ~ 0.461		
△angleβ	Sex	-0.993	-1.976 ~ -0.009	0.001	0.000
	Constant	27.367	25.766 ~ 8.968		
A ratio	Age	-0.005	-0.008 ~ 0.002	0.004	0.000
	Sex	-0.10	-.208 ~ -0.052	0.008	0.000
	Constant	4.181	3.971 ~ 4.391		

VPT, vibration perception threshold.; ABI, ankle-brachial index. CI, confidence interval; SDA, spatiotemporal variability; SDB, temporal variability.

### Comparison of gait parameters when sex and age were matched

**Figure 3:**
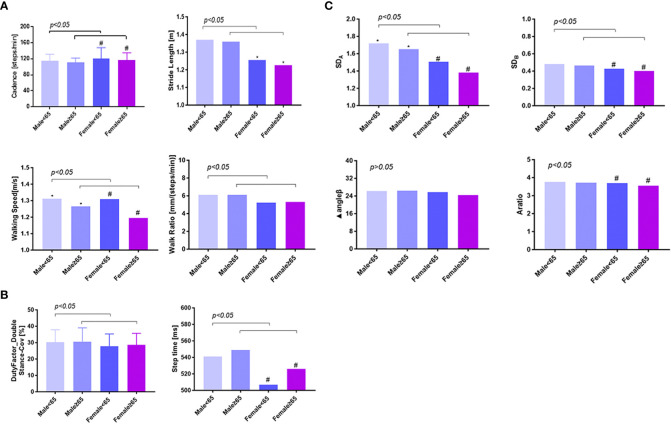
The gender and age difference of gait variables among type 2 diabetics. Panels **(A, B)** showed the comparison of spatiotemporal variables between different age and sex groups, and panel **(C)** showed the comparison of phase plot variables. A total of 1,420 individuals were selected and divided into four groups according to their sex and age and showed an obvious difference between men and women, young and elder patients. * *p* < 0.05, comparison between men of different age ranges; # *p* < 0.05, comparison between women of different age ranges; *p* < 0.05, comparison between different sex groups at the same age range. In the age range ≤65 years and ≥65 years, male participants were found to have fewer steps/min than female participants, but step length was significantly increased, walking faster, spent more time per walk, and showed greater SD_A_ and SD_B_. A larger part of distinction was observed only in female subjects, such as less steps/min, shorter stride length, more time spent per walk, and smaller SD_A_, SD_B_, and A ratio. Men<65 means men aged <65 years (56.1 ± 5.2 years), n = 372; Men≥65 means men aged ≥65 years (69.7 ± 3.2 years), n = 190; Women<65 means women aged <65 years (56.5 ± 5.8 years), n = 571; Women≥65 means women aged ≥65 years (69.7 ± 3.3 years), n = 287.

Based on the results above, the impact of sex and age on gait parameters needed to be clarified further, which was summarized in **Figures 3A–C**. In order to match the other factors between the groups, we selected 1,420 individuals and divided them into four groups according to their sex and age. Group 1 was defined as men, aged <65 years (56.1 ± 5.2 years), n = 372; Group 2, men, aged ≥65 years (69.7 ± 3.2 years), n = 190; Group 3, women, aged <65 years (56.5 ± 5.8 years), n = 571; Group 4, women, aged ≥65 years (69.7 ± 3.3 years), n = 287. As shown in **Figures 3A–C** and **Table 3**, male participants were found to have fewer steps/min than female participants in both ≤65 and ≥65 years of age (114.1 ± 0.8 steps/min vs. 120.9 ± 1.19 steps/min; 111.4 ± 0.58 steps/min vs. 116.7 ± 0.90 steps/min, *p* < 0.05), and the step length was significantly increased (1.37 ± 0.01 m/s vs. 1.25 ± 0.01 m/s; 1.36 ± 0.01 m/s vs. 1.23 ± 0.01 m/s, *p* < 0.05); they were walking faster (1.31 ± 0.01 m/s vs. 1.27 ± 0.01 m/s; 1.27 ± 0.01 m/s vs. 1.19 ± 0.01 m/s, *p* < 0.05), spent more time per walk (542.6 ± 2.07 ms vs. 518.3 ± 2.06 ms; 549.4 ± 2.42 ms vs. 526.1 ± 2.21 ms, *p* < 0.05), and showed greater SD_A_ (1.71 ± 0.02 vs. 1.48 ± 0.01; 1.66 ± 0.02 vs. 1.38 ± 0.02, *p* < 0.05) and SD_B_ (0.48 ± 0.01 vs. 0.42 ± 0.01; 0.47 ± 0.01 vs. 0.40 ± 0.01, *p* < 0.05). Moreover, male individuals had an increased walk ratio [6.10 ± 0.04 mm/(steps/min) vs. 5.33 ± 0.04 mm/(steps/min); 6.13 ± 0.05 mm/(steps/min) vs. 5.31 ± 0.04 mm/(steps/min), *p* < 0.05] and duty-factor_double stance (30.25% ± 0.36% vs. 28.1% ± 0.36%; 30.6% ± 0.49% vs. 28.56% ± 0.38%, *p* < 0.05). Elder male participants had significantly slower walking (1.27 ± 0.01 m/s vs. 1.31 ± 0.01 m/s, *p* < 0.05) and smaller SD_A_ (1.66 ± 0.02 vs. 1.71 ± 0.02, *p* < 0.05). A larger part of distinction was observed only in female subjects, such as less steps/min (116.7 ± 0.90 steps/min vs.120.9 ± 1.19 steps/min, *p* < 0.05), shorter stride length (1.23 ± 0.01 m vs. 1.25 ± 0.01 m, *p* < 0.05), more time spent per walk (526.1 ± 2.21 ms vs. 518.3 ± 2.06 ms, *p* < 0.05), and smaller SD_A_ (1.38 ± 0.02 vs. 1.48 ± 0.01, *p* < 0.01), SD_B_ (0.40 ± 0.01 vs. 0.42 ± 0.01, *p* < 0.05), and A ratio (3.55 ± 0.04 vs. 3.68 ± 0.04, *p* < 0.05).

**Table 3:** Comparison of dependent variables of gait characteristics among young and elder diabetic patients with different genders.

	Men<65	Men≥65	Women<65	Women≥65	*p*
Age (years)	56.1±5.2	69.7±3.2	56.5±5.8	69.7±3.3	–
Patients (n, %)	372,26.2%	190,13.4%	571,40.2%	287,20.2%	–
Cadence [steps/min]	114.1±0.8^a^	111.4±0.58^b^	120.9±1.19^a#^	116.7±0.90^b#^	<0.05
Stride length [m]	1.37±0.01^a^	1.36±0.01^b^	1.25±0.01^a#^	1.23±0.01^b#^	<0.05
Walking speed [m/s]	1.31±0.01^a*^	1.27±0.01^b*^	1.27±0.01^a#^	1.19±0.01^b#^	<0.05
Walk ratio [mm/(steps/min)]	6.10±0.04^a^	6.13±0.05^b^	5.33±0.04^a^	5.31±0.04^b^	<0.05
Duty Factor_Double Stance [%]	30.25±0.36^a^	30.6±0.49^b^	28.1±0.36^a^	28.56±0.38^b^	<0.05
Step time [ms]	542.6±2.07^a^	549.4±2.42^b^	518.3±2.06^a#^	526.1±2.21^b#^	<0.05
SD_A_	1.71±0.02^a*^	1.66±0.02^b*^	1.48±0.01^a#^	1.38±0.02^b#^	<0.05
SD_B_	0.48±0.01^a^	0.47±0.01^b^	0.42±0.01^a#^	0.40±0.01^b#^	<0.05
ΔAngle β	26.28±0.54	26.48±0.64	25.57±0.51	24.42±0.58	>0.05
A ratio	3.76±0.04	3.73±0.05	3.68±0.04^#^	3.55±0.04^#^	<0.05

Men<65 means men aged <65 years; Men≥65 means men aged ≥65 years; Women<65 means women aged <65 years; Women≥65 means women aged ≥65 years. Data were presented as mean ± SD or n (%) as appropriate.

*p < 0.05, Group 2 compared with Group 1; ^#^p < 0.05, Group 4 compared with Group 3; ^a^p < 0.05, Group 3 compared with Group 1; ^b^p < 0.05, Group 4 compared with Group 2. Data marked with the same letter indicate significant differences between groups.

### Discriminatory power

**Figure 4:**
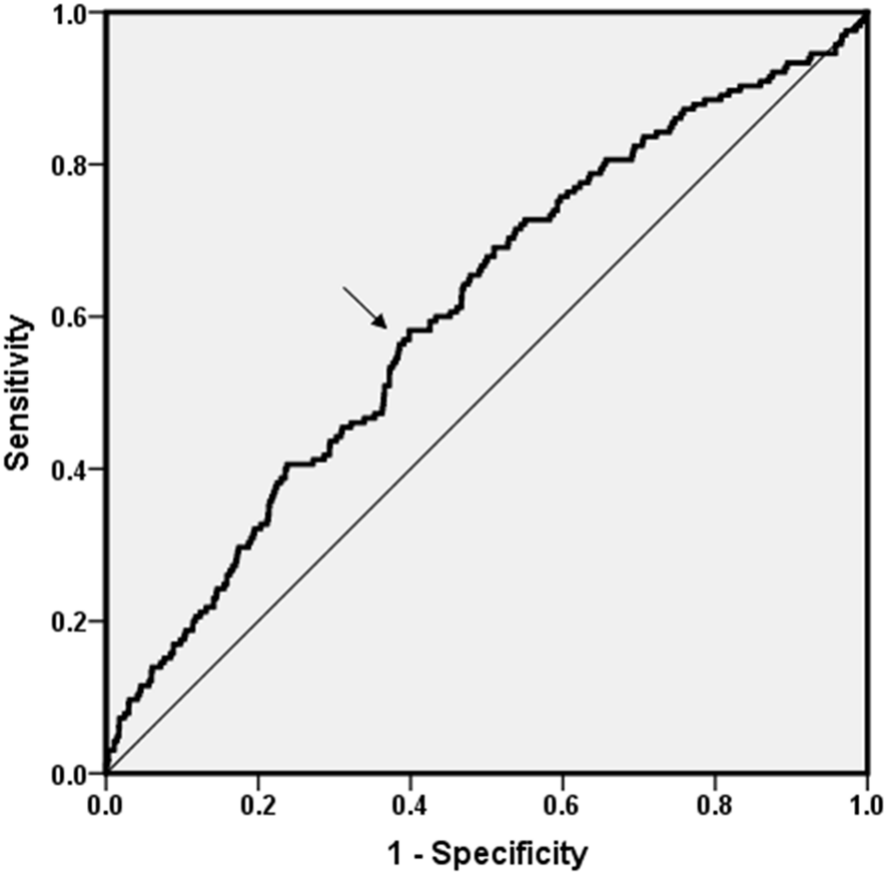
ROC curve analysis. ROC curve analysis was explored to find any discriminatory power of DPN for step time. The AUC value was 0.608 (95% CI: 0.562–0.654, *p* < 0.01). Cutoff point was 538.41 ms. The Youden index was 0.184; its sensitivity was 58.20% and the specificity was 39.81%.

Since step time was an independent correlative factor for DPN, ROC curve analysis was explored to find any discriminatory power and sensitivity and specificity of step time for the occurrence of DPN (**Figure 4**). The area under the curve (AUC) value was 0.608 (95% CI: 0.562–0.654, *p* < 0.01). In total, the cutoff point was 538.41 ms. The Youden index at this level was 0.184; its sensitivity was 58.20% and the specificity was 39.81%.

### Association of vibration perception threshold with increased step time

We chose an age range of NGT individuals from 46 to 75 years (average 56 years) as control and calculated the normal range of step time in the NGT elders (mean 527.32 ms, SD 45.01 ms). We used the mean ± twice the standard deviation as the normal range; >617.34 ms was classified as increased step time. As shown in **Table 4**, logistic regression analysis was used to analyze the relationship between VPT and increased step time, and there was a significant correlation between increased step time and increased levels of the highest VPT group (OR = 1.83, 95% CI: 1.32–2.55, *p* < 0.01). The OR value of female patients increased to 2.16 (OR = 2.16, 95% CI: 1.25–3.73, *p* < 0.01). This positive association persisted after adjustment for age (men: OR = 1.58, 95% CI: 1.02–2.46; women: OR = 2.17, 95% CI: 1.22–3.88, all *p* < 0.05).

Table 4Odds ratio analysis of VPT for increased step time.

**Table:** 

VPT (V)	Non-adjusted model						Age-adjusted model						
	Overall		Male		Female		Overall		Male		Female		
	OR (95%CI)	*p*	OR (95%CI)	*p*	OR (95%CI)	*p*	OR (95%CI)	*p*	OR (95%CI)	*p*	OR (95%CI)	*p*	
1~15	1	/	1	/	1	/	1	/	1	/	1	/
16~24	1.73 (0.85-3.55)	0.134	1.31 (0.50-3.38)	0.583	2.51 (0.84-7.53)	0.100	1.46 (0.71-.99)	0.301	1.08 (0.43-2.81)	0.881	2.08 (0.70-0.18)	0.186
≥25	1.83 (1.32-2.55)	0.000	1.63 (1.08-2.46)	0.021	2.16 (1.25-3.73)	0.006	1.82 (1.29-2.58)	0.001	1.58 (1.02-2.46)	0.042	2.17 (1.22-3.88)	0.009

**Table:** 

VPT (V)	Multiple factors-adjusted model												
	Overall				Male					Female			
	OR (95%CI)		*p*		OR (95%CI)			*p*		OR (95%CI)		*p*	
1~15	1		/		1			/		1		/	
16~24	1.001 (0.998-1.005)		0.368		0.999 (0.995-1.004)			0.797		1.003 (0.998-1.008)		0.187	
≥25	1.007 (1.003-1.011)		0.001		1.005 (1.000-1.011)			0.042		1.007 (1.001-1.014)		0.031	

Multiple factors-adjusted model was adjusted for other associated factors including age, leg length, and ABI.

OR, odds ratio; CI, confidence interval; VPT, vibration perception threshold.

## Discussion

Most recently, research has focused on the measurement of gait parameters to illustrate the role of diabetes, DPN, or other diabetes-related complications (2–6), while results were discrepant due to different populations, various devices and methods of examination, diverse analyses, and so on. For the purpose of verifying the effect of different conditions of diabetes on gait alterations during shod walking. The present study investigated the changes during walking among different diabetic individuals with controls for the first time and provided the first step of the whole diabetic population toward substantiating the impact of diabetes and diabetic lower extremity complications on gait.

Our results demonstrated that both the incidence of elevated blood glucose and the occurrence of lower extremity disease indicate the appearance of alterations during both spatiotemporal and phase plot analyses. Higher duty-factor_double stance-CoV was observed among individuals with elevated blood glucose. There are also other data available on the association between impaired blood glucose and abnormal gait. In a study by Almurdhi et al. (5), subjects with impaired glucose tolerance displayed a significantly higher dynamic mediolateral sway during walking, suggesting that alterations in gait may occur very early, even in the prediabetes phase. As these previous studies documented, the impairment of gait could be observed as early as the emergence of impaired glucose tolerance (IGT). This seems to suggest that gait abnormalities can be detected early, so the presence of gait abnormalities may help us detect diabetes early.

As documented in other studies (5, 6), diabetes alone could induce gait alterations, such as slower walking speed, shorter stride length, increased cadence, and high gait variability. In this study, there was no significant difference in walking speed and walk ratio among the four groups, which was inconsistent with some reports in Caucasians, Indians, Koreans, etc. We believe that these may be the following reasons: 1) Although the total sample size of this study was large, there was a large difference in sample size between groups, which may have contributed to the disappearance of the difference; 2) Walking speed and walk ratio can be compensated by walking posture, which also explains the changes of walking posture and gait status earlier than the changes of walking speed and walk ratio.

To our knowledge, there are no previous studies examining the alteration of phase plot within such an extended range of diabetes subjects. The trend analysis during phase plot assessment revealed that the decline of variability and symmetry appears as long as diabetes occurs. Many other investigations had offered explanations for abnormal gait features in diabetes. For instance, Almurdhi et al. (16) recently noted that diabetic patients had a significant reduction in proximal and distal leg muscle strength and a proximal reduction in muscle volume, with a further contribution of brain atrophy and cognitive impairments that were related with dysregulation of glycemic control (17). Some indications that a deprivation of nerve growth factors in subjects with diabetes have been demonstrated (18). Our findings in this pronouncedly larger sample of older adults contradict the results of previous studies concerning diabetes and gait in adults primarily older than 60 years. By doing so, it further enhances the insight into the relationship that gait deficits occurred as a consequence of diabetes.

It is widely recognized that DPN affects peripheral sensory and motor nerves (2) and then the sensorimotor system is gradually affected, resulting in a decreased sensation of pain, tissue damage, loss of muscle strength, changes in foot structure, and eventually abnormal gait (19). Lowered cadence, modified stride length, and decreased gait speed were reckoned as characteristics of an impaired gait performance among DPN groups (2). Contradicting to those results, the present study showed no significant difference in these parameters mentioned above but increased step time. Stepwise regression analysis revealed that VPT was an independent risk factor for decreased SD_A_ and SD_B_. All of these findings in our research demonstrated that DPN participants manifested a moderate modification of gait while walking to adapt sensorimotor system abnormality, much earlier than the presence of remarkable lower cadence, modified stride length, and decreased gait speed.

Several factors were considered to be responsible for the distinct results in our participants, such as sample size and control selection, 10-m level walking, eliminated process of acceleration and deceleration, and self-selected gait speed. Although the findings from previous studies were seemingly consistent (9, 20, 21), the sample size was strikingly small (approximately 50~100 cases), and usually, healthy individuals were picked as controls. However, the discrepancy disappeared as long as we extended the population to a huge sample and different diabetic complications. With respect to the impact of walking style demanded, the results varied apparently. It is meaningful to observe differences in various gait parameters while walking on challenging surfaces. For instance, Allet et al. (22) reported the difficulty of diabetic patients while changing from a tarred surface to cobblestones. Menz et al. (9) observed shorter step length of DPN patients when walking on irregular surfaces, and other evidence suggested that an irregular terrain accentuates differences in step time variability between older women with peripheral neuropathy and older women without peripheral neuropathy (23). Walking speeds (average 4.5–4.8 km/h) in the present study were within the range of values recorded in previous studies on comparable surfaces (3.4–5.1 km/h) (24) but showed no noticeable difference between groups. There was another trial that found a slower walking speed by patients with DPN when compared to control peers during the self-selected speed test (21). It has also been reported that both initiation and termination of gait were more complex procedures than steady-state walking (25). Except for all of these discussed reasons above, Gates et al. (26) noted that the sensory loss in these neuropathic patients was not complete; there are still retained proximal somatosensory inputs as well as visual and vestibular feedback information. Thus, we believe that there was no such difference in gait spatiotemporal analysis among various diabetic patients when performing a short-distance, non-weight-bearing, and level walking, even compared with non-DM ones.

Although majority of previous studies recruited DPN participants, only few studies excluded patients with LEAD particularly. In the present study, we excluded patients complicated by only LEAD in order to eliminate the impact of lower limb ischemia on the gait. In this large-sample and wide-range trial of elder individuals, we were able to examine potential explanatory factors (sex, age, BMI, height, leg length, complications, DM duration, diabetic comorbidities, smoking, VPT, and ABI) of the altered gait parameters. The key observation was that stepwise multivariate analyses identified that sex, age, and leg length were more significant independent predictors of gait parameters, in addition to VPT and ABI. In the present investigation, age-deteriorated changes existed almost in every gait feature. The elderly walked slower with lower cadence and shorter stride length as age increased. Slow walking speed is highly prevalent in men and women above age 65 years (27). Reduced walking speed appears to be a compensatory strategy adopted by the elders to maintain trunk stability, and it is associated with an increased risk of all-cause mortality, impaired gait efficiency, and an increased risk of disability (28). As de Mettelinge et al. (10) illustrated, older participants with diabetes walked slower, took shorter strides during simple, counting backward by 3 from 40, reciting animal names conditions when compared with controls, and showed more gait variability during dual-task conditions. Declined nervous system and musculoskeletal system because of aging may affect gait control (29). Age-deteriorated changes in the production of sex steroids and cortisol and in the secretion of the growth hormone and insulin-like growth factor-1 have also been identified in the pathogenesis of weakness during gait (30, 31). Sex-related changes in our investigation were popular too, which is consistent with other conclusions. As one South Korea trial described, women have a shorter stride length and walked slower than men mostly due to their shorter height, and the researchers assume that the difference is due to gender features of the gait-related anatomy and habits (32). The rapid reduction of estrogen in postmenopausal women and the gradual decline of testosterone in men lead to a decrease in muscle mass and strength (30). Moreover, we illustrated the influence of leg length on gait indexes, which should not be ignored during gait analysis. Decades ago, the locomotor advantages of longer lower limbs have been documented (33). Recently, 18 male healthy subjects were enrolled in a walking gait analysis (34), and Fazreena et al. maintained that the mean contact forces for all joints (ankle, knee, hip, and pelvis) in the short leg were increased. Researchers also claimed that gait impairments are associated with age, sex, diabetes, hypertension, and history of cerebrovascular accidents, of which greater age and female sex were listed to be associated with slower gait speed and shorter stride length (28).

In this investigation, ROC curve analysis was employed to seek the predictive value of step time in indicating the presence of DPN, and our research may be the first effort to find an optimal cutoff point of step time for predicting DPN (538.41 ms). However, the AUC was not so large enough for clinical practice, and we believe that the combined effect of different gait indexes, such as step time, stride length, and gait variability, must be more telling. Taking normal-VPT group as the referent, the positive association between increased step time and higher VPT group was observed in non-adjusted and age-adjusted models, and the association was more prominent in women, which means that women suffer from a much higher risk of increased step time than men.

There are some limitations in the present study that should be identified. This research was a multiple-center but cross-sectional analysis of outpatients with type 2 diabetes. Additional prospective studies are further required to determine the role of all diabetic conditions on the alterations of gait. This was a study of relatively older adults; therefore, the findings may not be suitable to assess younger samples or to individuals. In this study, plantar pressure could not be directly assessed but could only be indirectly analyzed by analyzing the foot status and gait of patients. Despite that this research was a multiple-center research, there is still a large difference in the number of people in different groups, which may be the reason why there is no difference in walking time, cadence, and stride length in this study. Although these limitations exist, we believe that the novel findings of the present research are generalizable to the large number of elder diabetic outpatients in the clinic.

## Conclusions

To date, current literature supports the role of DPN in altering gait parameters. However, the present investigation extended the participants to varied diabetic individuals, accompanied by lower extremity complications or not. We identified some significant differences in gait among different diabetic groups, such as an increased step time in DPN and lower SD_A_ and SD_B_ of subjects with DPN. We verified a close relation between VPT and gait alterations among elder Chinese individuals. Further hazard ratio and ROC curve analyses substantiate that the step time of walking was a simple and easier gait, which increases with worsening of DPN. Therefore, our study provides instructive significance of the impact of DPN on the alterations of gait, and the walking step time increases with the ascending VPT. The easily operated VPT screening is helpful to indicate the risk of abnormal walking mode and to prevent the fall-down, fracture, and even foot disorders in populations suffering from T2DM.

## Data availability statement

The raw data supporting the conclusions of this article will be made available by the authors, without undue reservation.

## Ethics statement

Written informed consent was obtained from the individual(s) for the publication of any potentially identifiable images or data included in this article.

## Author contributions

LD wrote and revised the article, YH and LX analyzed and processed data and helped to collect data, HZ and WS collected clinical data and gait data, PE and HD provided us with equipment and instructed us to use it, my professor FL provided research guidance and research fundings. All authors listed have made a substantial, direct, and intellectual contribution to the work, and approved it for publication.

## References

[1] Esser P Dawes H Collett J Feltham MG Howells K. Validity and inter-rater reliability of inertial gait measurements in parkinson's disease: A pilot study. J Neurosci Methods (2012) 205(1):177–81. doi: 10.1016/j.jneumeth.2012.01.005 22269595

[2] Mustapa A Justine M Mohd Mustafah N Jamil N Manaf H. Postural control and gait performance in the diabetic peripheral neuropathy: A systematic review. BioMed Res Int (2016) 2016:9305025. doi: 10.1155/2016/9305025 27525281 PMC4971307

[3] Esser P Dawes H Collett J Howells K. Insights into gait disorders: walking variability using phase plot analysis, parkinson's disease. Gait Posture (2013) 38(4):648–52. doi: 10.1016/j.gaitpost.2013.02.016 23510514

[4] Ritti-Dias RM Li J Hollabaugh KM Stoner JA Montgomery PS. V.O2 kinetics and clinical factors among patients with peripheral artery disease. J Cardiopulm Rehabil Prev (2013) 33(6):411–8. 10.1097/HCR.0000000000000025 PMC3888360 24189215

[5] Almurdhi MM Brown SJ Bowling FL Boulton AJM Jeziorska M. Altered walking strategy and increased unsteadiness in participants with impaired glucose tolerance and type 2 diabetes relates to small-fibre neuropathy but not vitamin d deficiency. Diabetes Med (2017) 34(6):839–45. doi: 10.1111/dme.13316 28103405

[6] Petrofsky J Lee S Macnider M Navarro E. Autonomic, endothelial function and the analysis of gait in patients with type 1 and type 2 diabetes. Acta Diabetol (2005) 42(1):7–15. doi: 10.1007/s00592-005-0168-0 15868108

[7] Pop-Busui R Ang L Boulton AJM Feldman EL Marcus RL Mizokami-Stout K. Diagnosis and treatment of painful diabetic peripheral neuropathy. Arlington (VA: American Diabetes Association (2022). 35544662

[8] Scarton A Guiotto A Malaquias T Spolaor F Sinigaglia G. A methodological framework for detecting ulcers' risk in diabetic foot subjects by combining gait analysis, a new musculoskeletal foot model and a foot finite element model. Gait Posture (2018) 60:279–85. doi: 10.1016/j.gaitpost.2017.08.036 28965863

[9] Menz HB Lord SR St George R Fitzpatrick RC. Walking stability and sensorimotor function in older people with diabetic peripheral neuropathy. Arch Phys Med Rehabil (2004) 85(2):245–52. doi: 10.1016/j.apmr.2003.06.015 14966709

[10] Roman de Mettelinge T Delbaere K Calders P Gysel T Van Den Noortgate N Cambier D. The impact of peripheral neuropathy and cognitive decrements on gait in older adults with type 2 diabetes mellitus. Arch Phys Med Rehabil (2013) 94(6):1074–9. doi: 10.1016/j.apmr.2013.01.018 23385112

[11] Allet L Armand S Golay A Monnin D de Bie RA de Bruin ED. Gait characteristics of diabetic patients: A systematic review. Diabetes Metab Res Rev (2008) 24(3):173–91. doi: 10.1002/dmrr.809 18232063

[12] Yavuzer G Yetkin I Toruner FB Koca N Bolukbasi N. Gait deviations of patients with diabetes mellitus: Looking beyond peripheral neuropathy. Eura Medicophys (2006) 42(2):127–33. 16767059

[13] Tominaga M. [Diagnostic criteria for diabetes mellitus]. Rinsho Byori (1999) 47(10):901–8. 10590663

[14] Raspovic A. Gait characteristics of people with diabetes-related peripheral neuropathy, with and without a history of ulceration. Gait Posture (2013) 38(4):723–8. doi: 10.1016/j.gaitpost.2013.03.009 23583607

[15] Cheung CL Lam KS Cheung BM. Diabetes is associated with increased risks of low lean mass and slow gait speed when peripheral artery disease is present. J Diabetes Complications (2016) 30(2):306–11. doi: 10.1016/j.jdiacomp.2015.11.015 26684167

[16] Almurdhi MM Reeves ND Bowling FL Boulton AJ Jeziorska M Malik RA. Reduced lower-limb muscle strength and volume in patients with type 2 diabetes in relation to neuropathy, intramuscular fat, and vitamin d levels. Diabetes Care (2016) 39(3):441–7. doi: 10.2337/dc15-0995 PMC5317239 26740641

[17] Cui X Abduljalil A Manor BD Peng CK Novak V. Multi-scale glycemic variability: A link to gray matter atrophy and cognitive decline in type 2 diabetes. PloS One (2014) 9(1):e86284. doi: 10.1371/journal.pone.0086284 24475100 PMC3901681

[18] Cameron NE Cotter MA. Potential therapeutic approaches to the treatment or prevention of diabetic neuropathy: Evidence from experimental studies. Diabetes Med (1993) 10(7):593–605. doi: 10.1111/j.1464-5491.1993.tb00131.x 8403819

[19] Vinik AI Strotmeyer ES Nakave AA Patel CV. Diabetic neuropathy in older adults. Clin Geriatr Med (2008) 24(3):407–35. doi: 10.1016/j.cger.2008.03.011 PMC2706706 18672180

[20] Sacco IC Amadio AC. A study of biomechanical parameters in gait analysis and sensitive cronaxie of diabetic neuropathic patients. Clin Biomech (Bristol Avon) (2000) 15(3):196–202. doi: 10.1016/S0268-0033(99)00060-1 10656981

[21] Camargo MR Barela JA Nozabieli AJ Mantovani AM Martinelli AR Fregonesi CE. Balance and ankle muscle strength predict spatiotemporal gait parameters in individuals with diabetic peripheral neuropathy. Diabetes Metab Syndr (2015) 9(2):79–84. doi: 10.1016/j.dsx.2015.02.004 25813140

[22] Allet L Armand S de Bie RA Pataky Z Aminian K Herrmann FR. Gait alterations of diabetic patients while walking on different surfaces. Gait Posture (2009) 29(3):488–93. doi: 10.1016/j.gaitpost.2008.11.012 19138520

[23] Richardson JK Thies SB DeMott TK Ashton-Miller JA. A comparison of gait characteristics between older women with and without peripheral neuropathy in standard and challenging environments. J Am Geriatr Soc (2004) 52(9):1532–7. doi: 10.1111/j.1532-5415.2004.52418.x 15341557

[24] Allet L Armand S de Bie RA Golay A Pataky Z Aminian K. Clinical factors associated with gait alterations in diabetic patients. Diabetes Med (2009) 26(10):1003–9. doi: 10.1111/j.1464-5491.2009.02811.x 19900232

[25] Grewal GS Bharara M Menzies R Talal TK Armstrong D Najafi B. Diabetic peripheral neuropathy and gait: Does footwear modify this association? J Diabetes Sci Technol (2013) 7(5):1138–46. doi: 10.1177/193229681300700506 PMC3876356 24124939

[26] Gates DH Dingwell JB. Peripheral neuropathy does not alter the fractal dynamics of stride intervals of gait. J Appl Physiol (1985) (2007) 102(3):965–71. doi: 10.1152/japplphysiol.00413.2006 PMC2827357 17110519

[27] Cummings SR Studenski S Ferrucci L. A diagnosis of dismobility–giving mobility clinical visibility: A mobility working group recommendation. JAMA (2014) 311(20):2061–2. doi: 10.1001/jama.2014.3033 PMC5012417 24763978

[28] Gardner AW Montgomery PS Casanegra AI Silva-Palacios F Ungvari Z Csiszar A. Association between gait characteristics and endothelial oxidative stress and inflammation in patients with symptomatic peripheral artery disease. Age (Dordr) (2016) 38(3):64. doi: 10.1007/s11357-016-9925-y 27273077 PMC5005916

[29] Park YS Kim JW Kwon Y Kwon MS. Effect of age and sex on gait characteristics in the Korean elderly people. Iran J Public Health (2018) 47(5):666–73. PMC6005965 29922608

[30] Chen X Mao G Leng SX. Frailty syndrome: An overview. Clin Interv Aging (2014) 9:433–41. 10.2147/CIA.S45300 PMC3964027 24672230

[31] Clegg A Young J Iliffe S Rikkert MO Rockwood K. Frailty in elderly people. Lancet (2013) 381(9868):752–62. doi: 10.1016/S0140-6736(12)62167-9 PMC4098658 23395245

[32] Cho SH Park JM Kwon OY. Gender differences in three dimensional gait analysis data from 98 healthy Korean adults. Clin Biomech (Bristol Avon) (2004) 19(2):145–52. doi: 10.1016/j.clinbiomech.2003.10.003 14967577

[33] Webb D. Maximum walking speed and lower limb length in hominids. Am J Phys Anthropol (1996) 101(4):515–25. doi: 10.1002/(SICI)1096-8644(199612)101:4<515::AID-AJPA6>3.0.CO;2-U 9016365

[34] Fazreena Othman N Salleh Basaruddin K Hanafi Mat Som M Shukry Abdul Majid M Razak Sulaiman A. The effect of leg length inequality on joint contact forces of lower limbs during walking. Acta Bioeng Biomech (2019) 21(1):55–62. 31197285

